# Assessment of genetic homogeneity of *in-vitro* propagated apple root stock MM 104 using ISSR and SCoT primers

**DOI:** 10.1186/s12870-024-04939-3

**Published:** 2024-04-03

**Authors:** Vandana Bisht, Janhvi Mishra Rawat, Kailash Singh Gaira, Sumit Purohit, Jigisha Anand, Somya Sinha, Debasis Mitra, Farid S. Ataya, Ahmed M. Elgazzar, Gaber El-Saber Batiha, Balwant Rawat

**Affiliations:** 1 G. B. Pant National Institute of Himalayan Environment, Kosi-Katarmla, Almora, 263643 Uttarakhand India; 2 Department of Biotechnology, Graphic Era Deemed to be University, Dehradun, 248002 Uttarakhand India; 3 G. B. Pant National Institute of Himalayan Environment, Sikkim Regional Centre, Pangthang, Gangtok, 737 101 Sikkim India; 4 Uttarakhand Council for Biotechnology, Pantnagar, U.S. Nagar, Haldi, 263145 Uttarakhand India; 5 Department of Microbiology, Graphic Era Deemed to be University, Dehradun, 248002 Uttarakhand India; 6 Department of Biochemistry, College of Science, King Saud University, PO Box 2455, Riyadh, 11451 Saudi Arabia; 7 Department of Veterinary Forensic Medicine and Toxicology, Alexandria University, Alexandria, Egypt; 8 Department of Pharmacology and Therapeutics, Damanhour University, Damanhour, AlBeheira Egypt; 9 School of Agriculture, Graphic Era Hill University, Dehradun, 248002 Uttarakhand India

**Keywords:** Fruit crop, Micro propagation, Molecular markers, Inter simple sequence repeats, Start Codon targeted, Genetic stability

## Abstract

Apple is an important fruit crop that is always in demand due to its commercial and nutraceutical value. Also, the requirement for quality planting material for this fruit crop for new plantations is increasing continuously. *In-vitro* propagation is an alternative approach, which may help to produce genetically identical high grade planting material. In this study, for the first time, an efficient and reproducible propagation protocol has been established for apple root stock MM 104 via axillary bud. Culturing axillary buds on Murashige and Skoog apple rootstock (MM 104) resulted in better *in-vitro* propagation. (MS) basal medium supplemented with 3.0% (w/v) sucrose and 0.8% (w/v) agar. The axillary buds were established in MS basal medium with BA (5.0 µM), NAA (1.0 µM) and further used to establish invitro propagation protocol. Plant Growth Regulators (PGRs), BA (1.0 µM) in combination with NAA (1.0 µM) was found most efficient for shoot multiplication (100%) and produced 9.8 shoots/explants with an average shoot length of (2.4 ± cm). All the shoots produced roots in 0.1 µM IBA with a 5-day dark period. Acclimatization of *in-vitro* raised plantlets was obtained with vermiculite: perlite: sand: soil (2:2:1:1) resulting in 76% survival under field conditions. The study showed that the use of axillary bud is efficient for multiple-shoot production of apple rootstock (MM 104). This is the first comprehensive report on *in-vitro* growth of apple root stock MM 104 with an assessment of genetic stability using DNA fingerprinting profiles based on Inter Simple Sequence Repeats (ISSR) and Start Codon Targeted (SCoT). The genetic stability of *in-vitro-*produced plants, as determined by SCoT and ISSR primers, demonstrated genetic closeness to the mother plant.

## Introduction

The apple is known as “the king of deciduous fruit” because of its lovely shape, attractive color, and delicious taste. It is a world-renowned table fruit that belongs to the family Rosaceae. Apple possesses some inherent characteristics such as high productivity, good shelf life, attractive appearance, and excellent flavor, which makes it a favorite fruit of the people. Since it can be grown only in a limited area in the hills while a large population in the plains of our country likes to enjoy this delicacy, it has tremendous scope for increasing its area and production [1].

Apple is one of the most promising fruit crops in the world, but India’s production is fairly low due to a variable environment [2]. It is mostly grown in the temperate Himalayan regions of Jammu & Kashmir, Himachal Pradesh, and Uttarakhand. On an area of 4.7 million acres, 75.4 million tonnes of apples are produced globally. China (47.7%), the United States (5.7%), India (3.8%), and Turkey (3.6%) are the world’s largest apple producers, in that order. Despite India’s third-place ranking in terms of output, its exports are still not up to par with its global standing [3]. The main causes of low production are a lack of high-quality and healthy planting materials, such as rootstock, and the size of ancient orchards.

Currently, the majority of commercial planting is becoming old and diseased and must be replaced. Micropropagation techniques have led the way for rapid plant multiplication and are effective in situations where quality planting material must be produced on a large scale and in a short period. Therefore, it is desirable to carry out clonal multiplication of improved rootstocks suited for the agro-climatic conditions of India’s apple-growing regions. Vegetative propagation is known for the production of true to type plants and will be useful to maintain the consistent genetic makeup of the apple fruit crop, as its genome is highly heterozygous [4–7].

Rootstock offers specific properties to the tree. They are of two types; seedling and clonal. Seedling rootstocks have the disadvantage of genetic variation, which leads to variability in the growth and performance of the scion of the grafted plant. Because of the above, clonal rootstocks have received increased attention as these are desirable, (i) to produce uniformity, (ii) to preserve special characteristics, (iii) to adapt to different climates, growth, and flowering habits and (iv) to maintain quality of fruit. To increase yield per unit area, the trend in apple production is moving towards higher-density planting. This trend was stimulated first by the availability of clonal size-controlling rootstocks, which permits denser plantings. Also, tissue culture raised plantlets of apple are free from any kind of contamination and prevents many plant health concerns. Tissue culture propagation is critical for facilitating international exchanges of apple cultivars and germplasm because tissue culture may be certified devoid of insects and phytopathogens than scion wood [8–10]. Thus, clonal rootstock multiplication via micropropagation aids in the commercial production of high-quality planting material in a short period that is suitable for the diverse agro-climatic conditions of apple-growing areas in India and has the characteristics of parent trees.

Apple rootstock MM 104 is semi-dwarf (60–75%, the size of trees on apple seedlings) and suitable for high-density planting. Due to its inherent precocity, this rootstock gained favor. It can perform well in mid-hill valleys with flat and irrigated soil, such as Kullu (1500–2100 amsl), Shimla, and the Union Territory of Jammu and Kashmir in India [11].

The ultimate aim and the most crucial part of plant tissue culture is to produce genetically similar (true-to-type) plants concerning their mother plant [12, 13]. Various molecular markers, such as RAPD, ISSR, SCoT, and others, can be used to determine genetic differences between regenerants and mother plants. These molecular markers are known for their sensitivity, dependability, and cost-effectiveness, and they are not impacted by environmental influences [14].

Although few studies on micropropagation of rootstock MM series have successfully developed using various explants [15–20], however, no investigations on the target species have been conducted about the planned objectives. The published reports do not include clonal fidelity analysis, which is a critical part of plant tissue culture practice.

The plus traits of MM 104 include its vigorous root system, increased productivity, starts bearing early, and show resistance to woolly aphids, heat and drought, and does not show sucker formation. It has good anchorage and tends to produce a more spreading tree [21]. Given these characteristics, the present study was undertaken to multiply MM 104 *in-vitro* and develop an improved propagation protocol so that the number of shoots/explants, plant height, rooting, and survival percentage of the rootstocks can be further augmented. Using Apple rootstock MM 104 as a model, this study was carried out to (1) construct an *in-vitro* propagation method for multiple shoot induction and (2) assess the genetic integrity of *in-vitro*-produced plants using SCoT and ISSR markers. The current study is the first thorough report on *in-vitro* propagation of Apple rootstock MM 104 employing direct organogenesis and genetic fidelity analysis.

## Results and discussion

### Sterilization

Successful micropropagation depends on the effectiveness of disinfection as well as the potential of surviving explants for regeneration. Conventional disinfestation methods were unsuccessful in controlling contamination. Therefore, the present study followed the two-step sterilization for the establishment of explants as follows (i) the twigs were washed with Tween- 20 and surface disinfected for 30 min in 0.1% HgCl_2,_ and (ii) After being removed from the aforementioned twigs, surface sterilization of the axillary buds was performed with 70% ethanol for 30 s, followed by disinfection with 0.1% HgCl_2_ solution for three minutes. This time duration was found optimum for twigs sterilization. The response of HgCl_2_ varied with the duration of treatment and concentration of HgCl_2_. It was observed that 0.2 to 0.5% (w/v) HgCl_2_ to sterilized MM 106 rootstocks axillary buds was best to remove contamination [22].

### Explant culture, shoot formation, and multiplication

After the sterilization process explants were cultured in a liquid medium initially. Serial transfer was most effective in the explant establishment of rootstock MM 104. This is reported to prevent the release of exudates and results in increased survival of explants [23]. Also, seasons play an important role in culture establishment. The best time to begin *in-vitro* culture with the least amount of contamination is in the spring season [24], although browning remains under control [25].

BA is regarded as one of the most effective cytokinins for plant growth and micropropagation [26], but response depends on concentration [27]. However, in the current study, the combination of BA and NAA showed the best result. The effect of PGRs in combination has also been reported in the apple rootstock MM 106 for shoot induction, however, the authors have used various combinations, such as, BAP, IBA, and GA_3_ [28, 29], BAP and IAA [30] and BAP, GA_3_, IBA, PVP and, PG [31] instead of NAA. Multiplication coefficient values of the present study were higher than the findings of some other researchers also [32].

MS medium supplemented with 5.0 µM BA and 1.0 µM NAA were used as multiplication media to obtain sufficient plant material for further experiments. Treatment 5 (1.0 µM BA with 1.0 µM NAA) showed a significant (*p* < 0.01) improvement compared to all other treatments regarding maximum shoots/explant (10 nos.), shoot length (2.4 cm), and shooting percentage (100%). The effect of treatment 12 (5.0 µM BA and 1.0 µM NAA) was also found significantly (*p* < 0.01) higher for rooting percentage (100%; Table 1).

**Table 1:** Effect of different concentrations and combinations of Plant Growth Regulators (BA and NAA) on shoot multiplication

Treatment No.	Concentration (µM)	Shoot number	Shoot length (cm)	Shoot regeneration (%)
T1	Control	2.93 (± 0.07)^de^	0.90 (± 0.08)^e^	64.08 (± 3.66)^d^
T2	1.0 BA	3.85 (± 0.08)^d^	1.23 (± 0.11)^d^	75.00 (± 7.22)^c^
T3	1.0 BA + 0.01 NAA	4.31 (± 0.09)^cd^	1.33 (± 0.09)^d^	95.83 (± 4.17)^ab^
T4	1.0 BA + 0.1 NAA	5.48 (± 0.13)^c^	1.65 (± 0.17)^bc^	91.67 (± 4.17)^b^
T5	1.0 BA + 1.0 NAA	9.83 (± 0.37)^a^	2.34 (± 0.13)^a^	100.00 (± 0.00)^a^
T6	2.5 BA	5.46 (± 0.11)^c^	1.46 (± 0.10)^c^	83.33 (± 4.17)^b^
T7	2.5 BA + 0.01 NAA	6.29 (± 0.20)^b^	1.78 (± 0.08)^ab^	75.00 (± 7.24)^c^
T8	2.5 BA + 0.1 NAA	5.32 (± 0.24)^c^	1.48 (± 0.18)^c^	87.50 (± 0.00)^b^
T9	2.5 BA + 1.0 NAA	7.11 (± 0.38)^b^	1.99 (± 0.01)^ab^	95.83 (± 4.17)^ab^
T10	5.0 BA	2.84 (± 0.26)^de^	1.51 (± 0.21)^c^	70.83 (± 4.16)^c^
T11	5.0 BA + 0.01NAA	2.50 (± 0.21)^de^	1.39 (± 0.16)^c^	91.67 (± 4.17)^b^
T12	5.0 BA + 0.1 NAA	2.88 (± 0.07)^de^	1.47 (± 0.11)^c^	100.00 (± 0.00)^a^
T13	5.0 BA + 1.0 NAA	5.71 (± 0.36)^c^	2.29 (± 0.42)^a^	95.83 (± 4.17)^ab^
T14	10.0 BA	2.26 (± 0.30)^de^	1.56 (± 0.11)^c^	75.00 (± 7.22)^c^
T15	10.0 BA + 0.1 NAA	2.47 (± 0.20)^de^	1.73 (± 0.13)^ab^	70.83 (± 4.17)^c^
T16	10.0 BA + 1.0 NAA	2.94 (± 0.15)^de^	1.43 (± 0.10)^c^	75.00 (± 0.00)^c^
T17	1.0 NAA	1.20 (± 0.00)^f^	0.86 (± 0.00)^e^	56.66 (± 0.00)^e^

Data are mean of three replicates each with eight explants, values in parenthesis are ± SE For each parameter, and values with different letters in superscript are significantly different (*p* < 0.05)

**Fig. 1:**
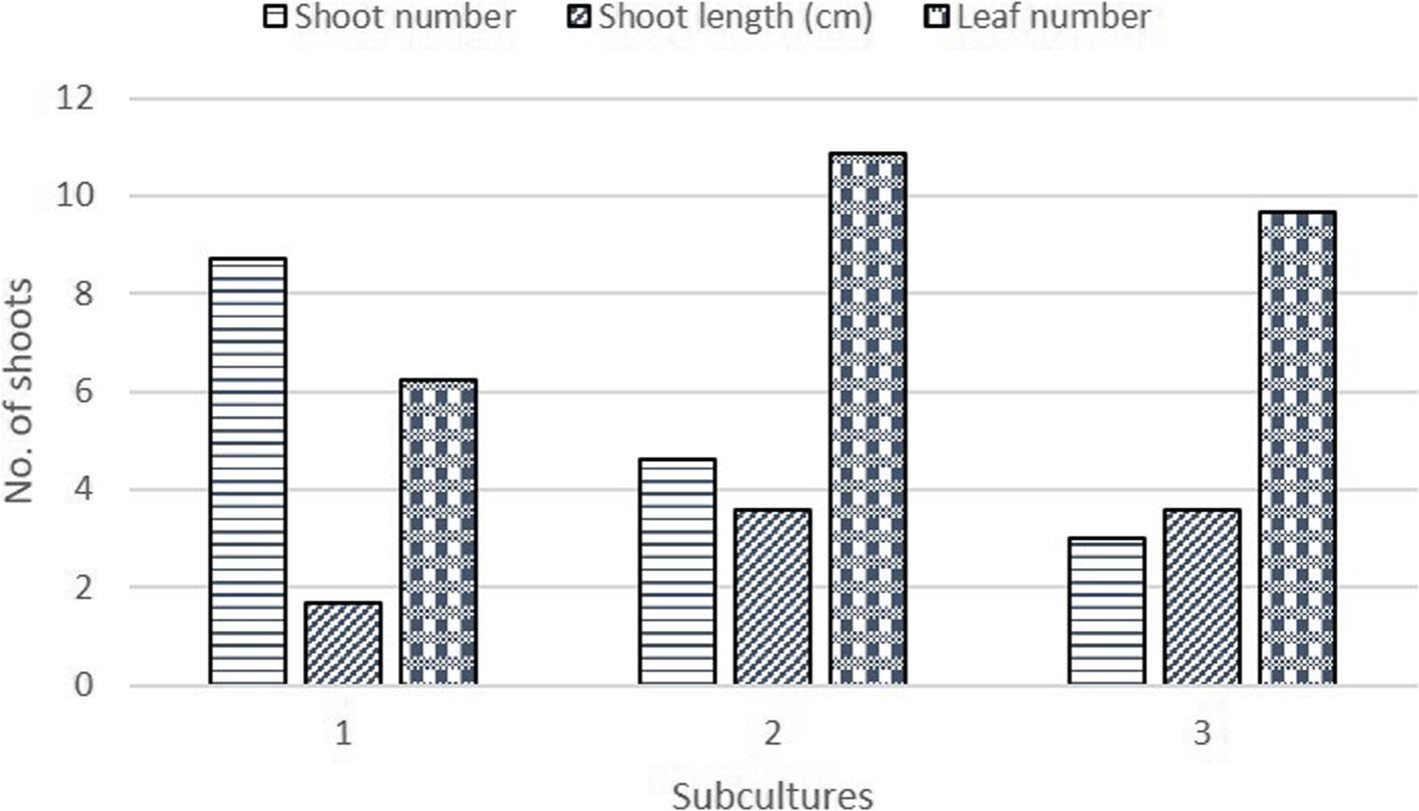
Effect of subculture (weeks) on shoot multiplication of MM 104

To find out the effect of subculture on shoot multiplication, the best-responding media (1.0 µM BA and 1.0 µM NAA) of shoot multiplication was used. Results showed that subculturing was not found suitable for the number of new shoots as shoot numbers decreased with subculture duration (Fig. 1). However, for obtaining longer shoots and a greater number of leaves, subcultures II and III were found suitable, as shoot length and leaf number increased significantly (*p* < 0.01) with subculture (II, and III) duration (Fig. 1).

### Rooting under in-vitro condition

Types of auxin and its concentration in the medium play a critical role in rooting response of *in–vitro-*regenerated shoots. Several researchers reported the effect of strength of MS media (half strength, one third and, one fourth strength) and auxin types has on the rooting of *in-vitro* regenerated plantlets of apple [20, 32]. Also, favorable effect of a diluted mineral solution on rooting is reported, which could be due to reduction in nitrogen concentration in the media [33].

**Fig. 2:**
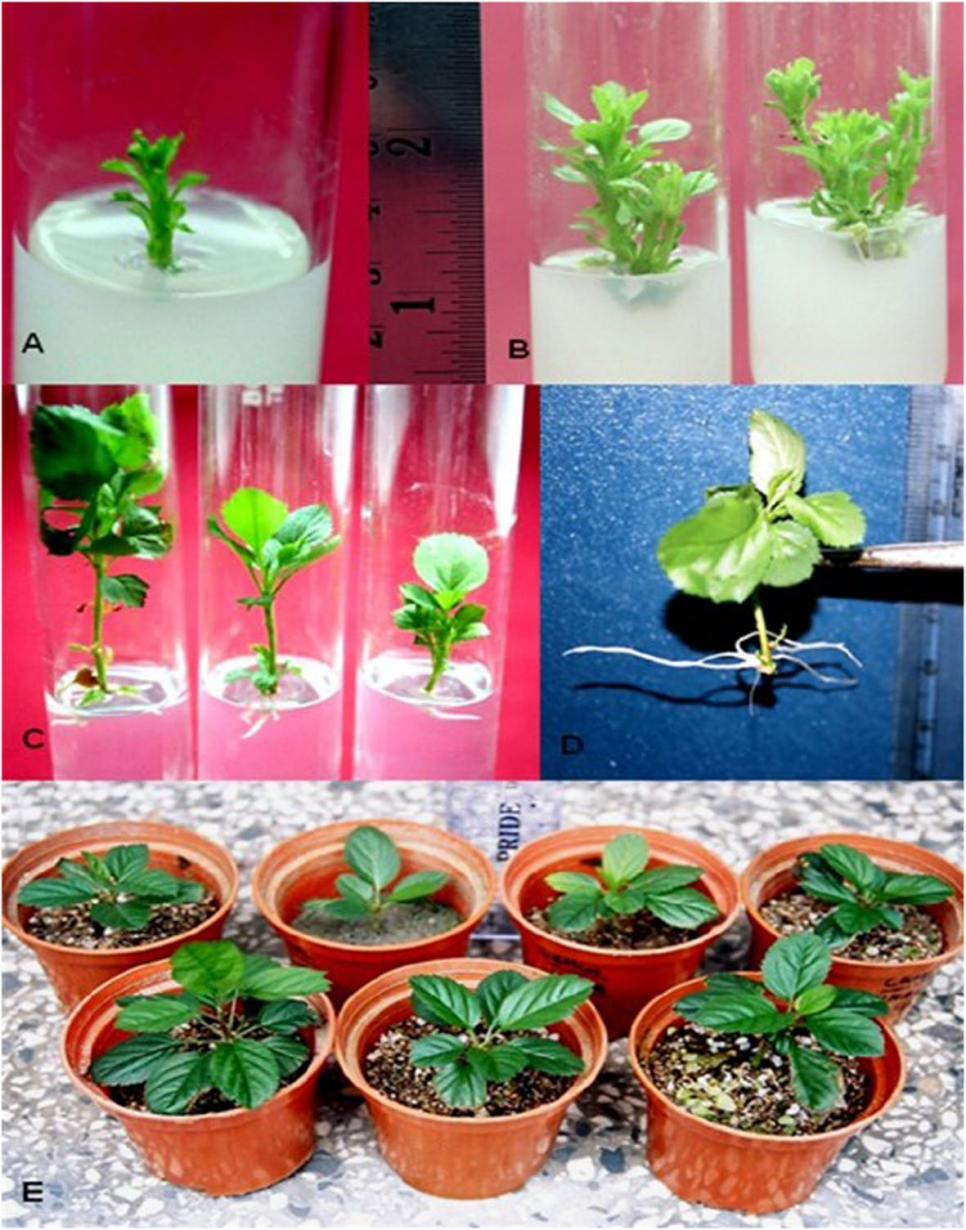
*In-vitro* propagation of apple rootstock “MM 104” through the axillary bud. **A** Shoot induction on 5.0 µM BA and 1.0 µM NAA; **B** Shoot multiplication on 1.0 µM BA and 1.0 µM NAA; **(C)** Root induction in 0.01 µM IBA; **D** Root elongation in 0.1 µM IBA; **E ***In-vitro* raised plantlets after 4 weeks under ex vitro conditions

To identify the best conditions for rooting, a detailed experiment was conducted. Auxin type and concentration play a critical role in the rooting responses of *in-vitro-*generated shoots. Two auxin types with different concentrations were tested. IBA was found more effective for rooting, which is similar to the previous study [17]. The treatment (IBA concentrations) effect was observed highly significant (*p* < 0.01) for the number of roots, length of root, and rooting percent. The effect of seven days of dark duration for the number of roots, length of root, and percentage of rooting was found significant (*p* < 0.05). Similarly, the interactive effect of treatment and duration was found significant (*p* < 0.05) for number of number of roots, length of root and rooting percent. The comparative performance reveals that the low concentration of IBA (0.1 µM) and 7-day dark period was found best for maximum rooting (91.7%) and longest root (3.81 cm) significantly (*p* < 0.01) (Fig. 2C, D; Table 2). A maximum number of roots (7.48 roots/shoot) was obtained by 1.0 µM IBA and 1 µM NAA in combination without dark treatment. Only 10% rooting with NAA and 20–25% IBA were reported in the previous study [17], when shoots were directly transferred to rooting media. The present study, however, shows very high rooting (91.7%) due to the initial seven days of dark duration. The rooting rate in the present study is higher than the previous studies [16, 34].

**Table 2:** Effect of different IBA concentration and dark duration treatment on rooting of MM 104 shoots

Concentration (µM)	Dark duration (days)	Root number	Root length (cm)	Rooting %
0.01 IBA	0	1.42 (± 0.22)^ef^	1.68 (± 0.28)^b^	50.00 (± 14.43)^d^
	1	1.75 (± 0.14)^e^	0.78 (± 0.16)^d^	41.67 (± 8.33)^e^
	5	2.33 (± 0.17)^e^	1.90 (± 0.05)^a^	33.33 (± 8.33)^f^
	10	2.52 (± 0.48)^de^	2.13 (± 0.81)^a^	75.00 (± 7.22)^b^
	15	4.27 (± 0.93)^c^	2.04 (± 0.20)^a^	70.83 (± 4.17)^b^
0.1 IBA	0	2.11 (± 0.31)^e^	1.82 (± 0.22)^ab^	29.17 (± 4.17)^f^
	1	1.72 (± 0.15)^e^	1.79 (± 0.17)^b^	29.17 (± 4.17)^f^
	5	4.25 (± 0.73)^c^	1.70 (± 0.30)^b^	100.00 (± 0.00)^a^
	10	2.78 (± 1.12)^e^	1.36 (± 0.25)^c^	58.33 (± 16.67)^c^
	15	2.19 (± 0.10)^e^	1.20 (± 0.30)^c^	33.33 (± 11.02)^f^
1.0 IBA	0	3.14 (± 0.34)^d^	0.58 (± 0.06)^d^	66.67 (± 8.33)^bc^
	1	4.64 (± 1.08)^bc^	0.58 (± 0.06)^d^	41.67 (± 4.17)^e^
	5	6.23 (± 1.02)^a^	0.78 (± 0.17)^d^	54.17 (± 4.17)^cd^
	10	4.67 (± 1.09)^bc^	0.63 (± 0.10)^d^	58.33 (± 8.33)^c^
	15	5.83 (± 0.42)^a^	0.37 (± 0.09)^e^	45.3 (± 15.02)^e^
5.0 IBA	0	4.00 (± 0.00)^c^	0.73 (± 0.19)^d^	33.33 (± 11.02)^f^
	1	2.17 (± 1.09)^e^	0.25 (± 0.14)^f^	12.50 (± 7.22)^h^
	5	1.67 (± 0.88)^e^	0.20 (± 0.12)^f^	8.33 (± 4.17)^i^
	10	4.11 (± 1.16)^c^	0.32 (± 0.06)^f^	29.17 (± 4.17)^fg^
	15	2.00 (± 2.00)^e^	0.15 (± 0.15)^g^	8.33 (± 8.33)^i^
10.0 IBA	0	2.17 (± 1.17)^e^	0.37 (± 0.19)^f^	12.50 (± 7.22)^h^
	1	5.33 (± 2.91)^ab^	0.27 (± 0.15)^f^	8.33 (± 4.17)^i^
	5	1.50 (± 0.76)^e^	0.30 (± 0.17)^f^	12.50 (± 7.22)^h^
	10	5.00 (± 1.00)^b^	0.38 (± 0.06)^f^	25.00 (± 12.50)^g^
	15	3.33 (± 1.76)^d^	0.43 (± 0.23)^f^	8.33 (± 4.17)^i^

Data are mean of three replicates each with eight explants, values in parenthesis are ± SE. For each parameter, values with different letters in superscript are significantly different (*p* < 0.05)

The above experiment shows that out of two auxins (IBA and NAA) IBA was found best and out of three concentrations of IBA (0.1, 1.0, 10.0 µM) 0.1 IBA with 7 days dark period was best for rooting. With this experiment, we have tried to optimize the concentration of IBA and dark duration. The concentration of IBA (treatments), dark duration and interactive (treatment and duration) effects were found significant (*p* < 0.01) for rooting percentage (Table 2). While only the treatments effect was found significant (*p* < 0.01) for root number, root length and percent rooting. It was noticed that lowering the IBA concentration from 0.1 to 0.01 did not improve the rooting percentage. But by lowering the dark duration to 5 days from 7 days, 100% rooting was achieved. Higher concentrations (5.0 and 10.0 µM) of IBA were not suitable for percent rooting and root length, though the number of roots was increased with higher concentrations of IBA [35–37].

### Acclimatization

Well-rooted shoots were used for ex-vitro transfer from different substrate types and combinations. Maximum survival rate (76%) was obtained with substrate type Vermiculite: Perlite: Sand: Soil (2:2:1:1; Fig. 3). The treatment (substrates) effect was significant (*p* < 0.05) for survival percentage, and non-significant for shoot growth. The ratio of substrates also plays an important role in improving the survival rate of ex- vitro plants. During the *ex-vitro* transfer, fungal infection was a major problem, therefore, all the plants were treated with Bavistin (fungicide; 0.1% w/v) before their transfer to the green house. This intervention reduced infections and improved the survival rate. The current study’s *ex-vitro* survival rate (76.0) was higher than the prior report’s (70%) [17].

**Fig. 3:**
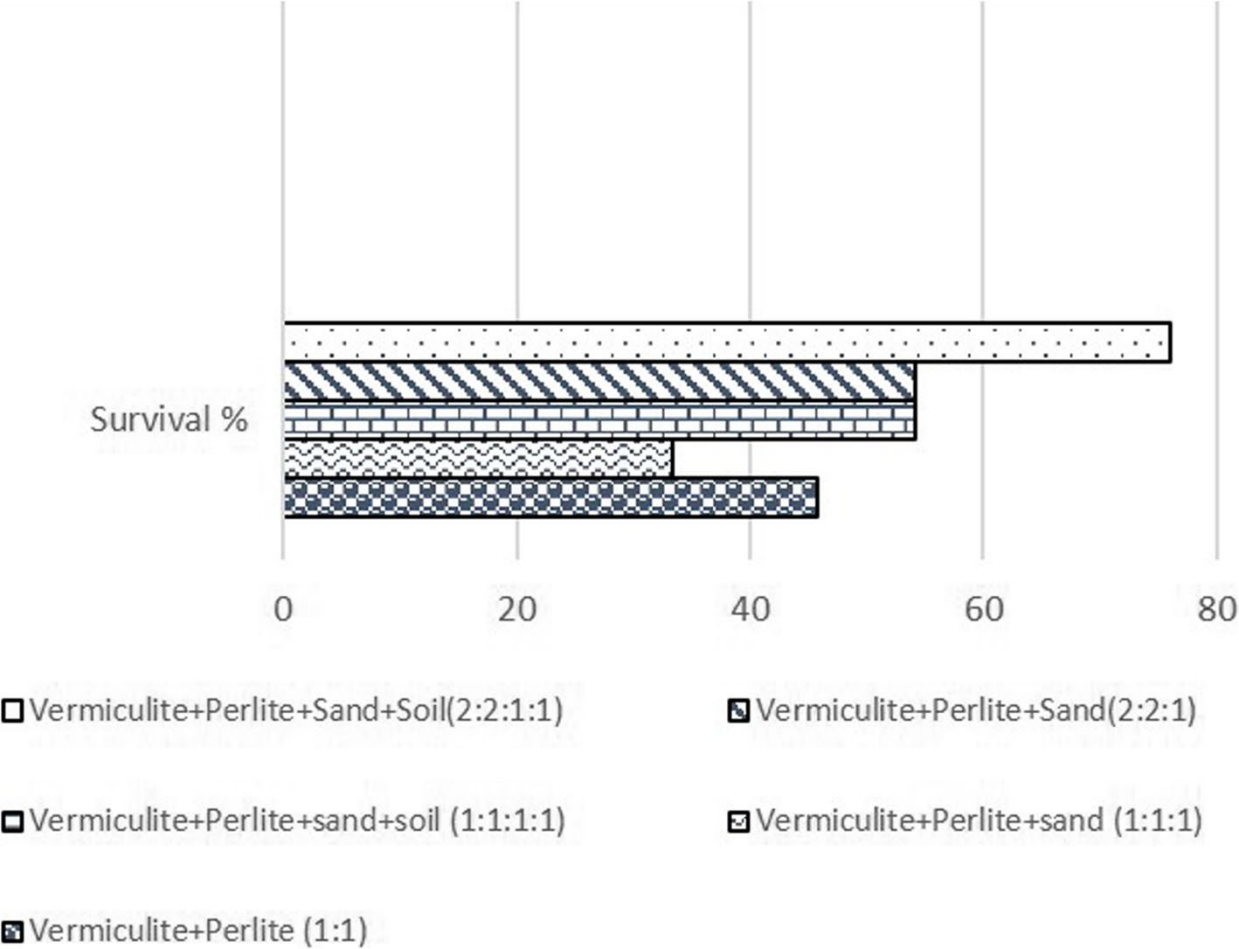
Effect of substrate type on survival % of *in-vitro-*regenerated plants of apple root stock MM 104

For *in-vitro* propagation of apple rootstock (MM 104) reports of various explants and PGRs used with different combinations and concentrations, have shown different results. Compared to the results of *in-vitro* propagation of previous researchers, the present protocol is an improved one regarding shoot multiplication (100% vs. 93.3%), number of shoots produced per explant (9.8 shoot/explant vs. 6.66), rooting percentage (100% vs. 80–90% using axillary bud) and survival rate at the field conditions (76% vs. 70%). The subculture duration of the previous study (7–8 weeks) [17] is higher than the present study (4 weeks).

### Genetic fidelity analysis

Somaclonal diversity developed in micro-propagated plants as a result of distinct explant sources, different culture circumstances, media component imbalances caused by high phytohormone concentration, different regeneration procedures, and extended sub-cultural passage [12, 38, 39]. As a result, when primary regenerants are chosen as commercial end products, evaluating somaclonal variation to assess the genetic stability of micropropagated plants is critical [40, 41].

ISSR and SCoT markers were utilized in this work to investigate the likelihood of any genetic variation that may be triggered by the physical or chemical parameters in the culture medium. Since micropropagation causes somaclonal variation in micropropagated plants, utilizing multiple markers has long been recommended for a comprehensive investigation of plant genetic homogeneity [42, 43]. ISSR investigations use non-coding regions of DNA to investigate genetic diversity, variability, and stability. The unique and gene-targeted molecular marker technique known as SCoT, is believed to be more accurate in determining genetic homogeneity because it was created from flanking ATG translation codons in plant genes [43–45]. The multilocus feature of the marker aids in determining high genetic polymorphism. Furthermore, lengthy primers and high annealing temperatures improve the reproducibility of SCoT primer [43, 46]. SCoT markers appear frequently in the genome and give considerable genetic information since they are associated with initiation codons [47, 48].

A total of 36 ISSR primers were screened for the analysis, of which 10 ISSR primers produced 64 reproducible bands ranging from 100 to 2700 bp, with an average of 6.4 bands per primer. Maximum 8 monomorphic bands were produced by ISSR primer 849, followed by primer 801, primer 830, and primer 845, whereas, minimum 5 monomorphic bands were produced by primer 808 and 841. Out of 64 amplified products 63 bands were monomorphic (98.43%; Table 3). This shows a high degree of monomorphism between control and invitro regenerants. Similar results of low polymorphism and high genetic identity have also been reported by several workers [12, 38, 41].

**Table 3:** Genetic fidelity analysis of invitro regenerated plants of apple root stock MM 104 using ISSR and SCoT marker

Primers used (ISSR and SCoT)	Primer sequence (5’ to 3’)	No. of scorable bands		No. of bands		Percentage		Band size (kb)
				Monomorphic	Polymorphic	Monomorphism	Polymorphism	
ISSR 801	(AT)8 T	7		7	-	100	-	2.5–0.5
ISSR 803	(AT) 8 C	6		6	-	100	-	1.5–0.5
ISSR 808	(AG) 8 C	5		5	-	100	-	2.0–0.5
ISSR 811	(GA) 8 C	5		4	1	80	20	2.0–0.5
ISSR 828	(GT)8 A	8		8	-	100	-	2.5–0.2
ISSR 830	(TG) 8 G	7		7	-	100	-	2.0–0.1
ISSR 841	(GA)8 YC	5		5	-	100	-	2.7–0.2
ISSR 845	(CT) 8 RG	7		7	-	100	-	2.5–0.5
ISSR 848	(CA)7CRG	6		6	-	100	-	2.5–0.2
ISSR 849	(GT)8 YA	8		8	-	100	-	2.5–0.2
SCoT1	CAACAATGGCTACCACCA		4	4	-	100	-	1.5–0.2
SCoT 2	CAACAATGGCTACCACCC		5	5	-	100	-	1.8–0.5
SCoT 4	CAACAATGGCTACCACCT		4	4	-	100	-	1.5–0.5
SCoT 5	CAACAATGGCTACCACGA		8	7	1	87.5	12.5	1.5–0.2
SCoT 8	CAACAATGGCTACCACGT		4	3	1	75	25	1.5–0.2
SCoT 9	CAACAATGGCTACCAGCA		3	3	-	100	-	1.5–0.5
SCoT 12	ACGACATGGCGACCAACG		5	5	-	100	-	2.0–0.3
SCoT 15	ACGACATGGCGACCGCGA		9	9	-	100	-	1.5–0.2
SCoT 16	ACCATGGCTACCACCGAC		3	3	-	100	-	1.5–0.2
SCoT 24	CACCATGGCTACCACCAT		4	4	-	100	-	1.5–0.2
SCoT 26	ACCATGGCTACCACCGTC		10	10	-	80	20	1.5–0.2
SCoT 33	CCATGGCTACCACCGCAG		5	5	-	100	-	1.5–0.2

**Fig. 4:**
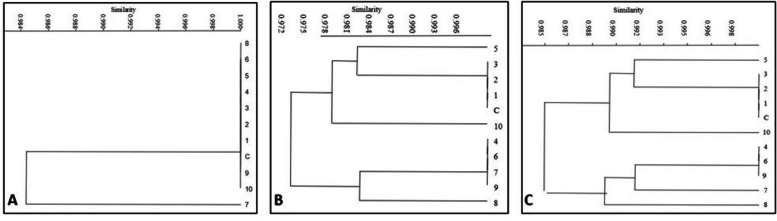
Banding profiles obtained for *in-vitro-*raised plants from apple root stock MM 104. **A** ISSR profile with primer 848 (**B**) SCoT profile for with primer 8. Where, M is 1 kb molecular weight marker; C is Control Plant and 1 to 10 randomlyselected in vitro regenerated plants of apple

SCoT markers, which identified genetic variation based on the brief conserved area flanking the ATG start codon in plant genes, were used to further confirm the results of ISSR analysis. A total of 12 primers produced 64 unambiguous and repeatable bands with an average of 5.3 bands per primer and band sizes ranging from 200 to 2000 bp following an initial screening with 21 SCoT primers (Table 4). The SCoT primer 26 produced the most bands (10), followed by the SCoT 15 primer (9), and the SCoT 9 and SCoT 16 primers produced the fewest bands (3). Representative gel photographs of ISSR and SCoT markers are presented in Fig. 4.

**Table 4:** Summary of ISSR and SCoT amplified products

Description	ISSR	SCoT
Total bands scored	64	64
Number of monomorphic bands	63	60
Number of polymorphic bands	1	4
Number of primers used	10	12
Average polymorphism per primer	0.1	0.33
Average number of fragments per primer	6.4	5.3
Size range of amplified fragments (kb)	0.1–2.7	0.2–2.0

The results showed that the *in-vitro* regenerants and control plant had excellent genetic uniformity (93.75%) and minimal genetic variation (6.25%). Similar findings of low polymorphism among the micropropagated plants were found in an earlier work utilizing the SCoT marker [41]. Low polymorphism (3.90%) and great genetic stability (96.10% monomorphism) were found in the micropropagated plants, according to the combined ISSR and SCoT analysis results, which revealed 123 monomorphic and 5 polymorphic bands from a total of 128 scorable bands. The somaclonal changes in the *in-vitro*-regenerated plants may be responsible for the current low degree of polymorphism seen through the ISSR (1.57%) and SCoT (6.25%) study.

### Genetic identity, genetic distance, and cluster analysis

**Fig. 5:**
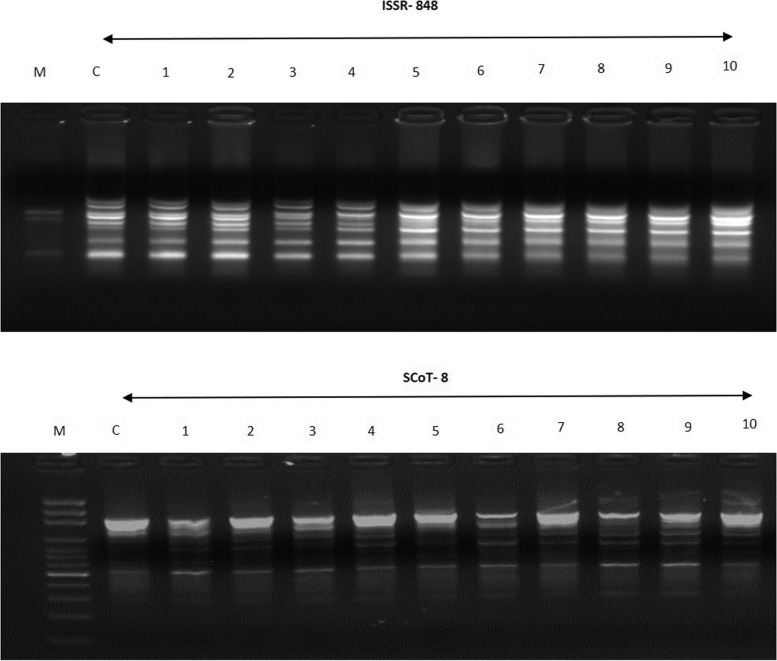
Dendrogram of ISSR (**A**), SCoT (**B**) and Pooled ISSR-SCoT (**C**) showing the genetic relationship between controlplant (**C**) and ten randomly selected *in-vitro-*regenerated plants (1 to 10) of apple from root stock MM104

Nei’s genetic distance values were 0.000 and 0.015 in a genetic distance matrix created from the combined ISSR-SCoT data, and Nei’s genetic identity values were 0.984 and 1.000, showing significant genetic identity between the regenerants and the control plant. The genotypes of 7 differed from all the *in-vitro*-regenerated plants, including the control plant, by a tight Nei’s genetic distance matrix value of 0.015 each, whereas *in-vitro*-regenerated 7, 8, 9, and 10 differed from 1, 2, 3, 4, 5, 6, and control plant by an identical matrix value of 0.948. The investigation also showed that the *in-vitro*-grown plants 1, 2, 3, 4, 5, and 6 had a complete genetic homogeneity of (0.000 or 1.000; Table 5). Numerous researchers have also documented the observed genetic homogeneity between the *in-vitro*-regenerants and the mother plant [12, 41]. Two clusters were found using the ISSR marker in Nei’s genetic distance-based dendrogram between the *in-vitro* regenerants and the control plant (Fig. 5A). The control plant and every other *in-vitro-*grown plant, except for sample number 7, displayed a single large cluster in the ISSR-based dendrogram (Fig. 5A). The *in-vitro*-regenerants 1, 2, 3, and control plant were clustered together in the dendrogram analysis of SCoT, while 4, 6, 7, and 9 formed another cluster. Sample 10 was placed by itself in a different cluster (Fig. 5B).

**Table 5:** Nei’s genetic identity (above diagonal) and genetic distance (below diagonal) matrices between control plant (c) and *in-vitro* regenerants (1–10) of apple (rootstock MM104) based from pooled data of ISSR and SCoT analysis

	C	1	2	3	4	5	6	7	8	9	10
**C**	****	1.000	1.000	1.000	1.000	1.000	1.000	0.984	1.000	1.000	1.000
**1**	0.000	****	1.000	1.000	1.000	1.000	1.000	0.984	1.000	1.000	1.000
**2**	0.000	0.000	****	1.000	1.000	1.000	1.000	0.984	1.000	1.000	1.000
**3**	0.000	0.000	0.000	****	1.000	1.000	1.000	0.984	1.000	1.000	1.000
**4**	0.000	0.000	0.000	0.000	****	1.000	1.000	0.984	1.000	1.000	1.000
**5**	0.000	0.000	0.000	0.000	0.000	****	1.000	0.984	1.000	1.000	1.000
**6**	0.000	0.000	0.000	0.000	0.000	0.000	****	0.984	1.000	1.000	1.000
**7**	0.015	0.015	0.015	0.015	0.015	0.015	0.015	****	0.984	0.984	0.984
**8**	0.000	0.000	0.000	0.000	0.000	0.000	0.000	0.015	****	1.000	1.000
**9**	0.000	0.000	0.000	0.000	0.000	0.000	0.000	0.015	0.000	****	1.000
**10**	0.000	0.000	0.000	0.000	0.000	0.000	0.000	0.015	0.000	0.000	****

The pooled ISSR-SCoT data’s dendrogram revealed two distinct clusters. The control plant and *in-vitro*-regenerants 1, 2, 3, and 4 were grouped in one large cluster, but *in-vitro*-regenerants 4, 6, 9, and 7 were present in another cluster, indicating their genetic divergence from the other genotypes (Fig. 5C). According to multiple researchers [12, 41], the cause of this could be somaclonal changes caused by the presence of various chromosomal defects in *in-vitro*-regenerated plants.

**Fig. 6:**
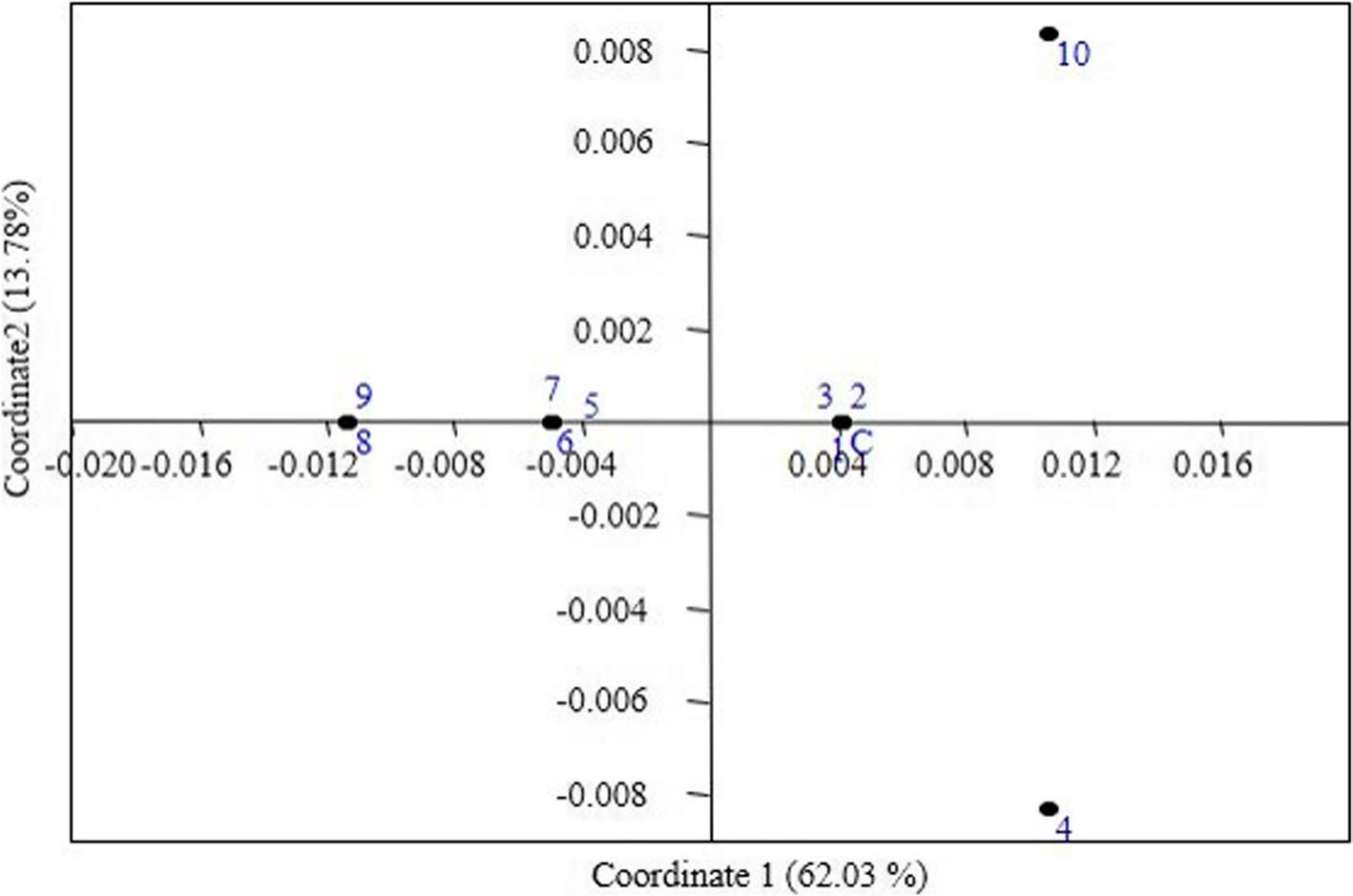
PCoA plot depicting the distribution of the control plant (C) and ten *in-vitro-raised* plants (1 to 10) of apple obtained from pooled SSR-SCoT data

The spatial distribution of genotypes 1, 2, 3, 4, and 10 including the control plant was found in the first and fourth quadrants, according to two-dimensional principal coordinate analysis (PCoA) of pooled data sets of ISSR and SCoT markers, whereas genotypes 5, 6, 7, 8 and 9 were nested together in the third quadrant (Fig. 6). The dendrogram-generated clustering pattern was nearly identical to the grouping pattern of the mother plant and the regenerants.

## Conclusion

This is the first account of genetic fidelity investigation in apple rootstock MM 104 plants that were grown under *in-vitro* condition. The study also revealed an enhanced micropropagation strategy for this species using axillary buds, which will give an alternate means of supplying high-quality planting material for commercial application. We showed the genetic homogeneity and true-to-type nature of *in-vitro-*regenerated plants using SCoT and ISSR markers. Such a strategy can be used to provide superior planting material that will aid in increasing crop productivity.

The following conclusions can be drawn from the present study: (i) the current study offers an enhanced, effective, and manageable tissue culture methodology for mass multiplication of apple rootstock MM 104 (ii) application of this protocol for providing large-scale planting material can be helpful to adopt the high-density planting in Uttarakhand hills, (iii) detailed rooting experiments improved the rooting rate and provide 100% rooting in a very low concentration of auxin, (iv) *in-vitro-*raised plantlets of rootstock MM 104 showed genetic similarity and optimal survival under ex-vitro conditions after simple acclimatization procedure. Adoption of such techniques by different stakeholders will boost apple production in long-term and the development of reproducible *in-vitro* regeneration protocol for rootstock MM 104 through axillary bud would facilitate undertaking genetic transformation studies for further improvement of this rootstock. The differentiation of the cultivars using molecular markers such as ISSR and SCoT, may add helpful insights into the cultivars’ genetic diversity and provide essential knowledge for their selection as genetic resources in breeding new cultivars.

### Methods

#### Explants

Twigs (10–15 cm) of rootstock MM 104 were collected from the Horticulture Garden, situated at Chaubattia, Ranikhet (29^0^36’55” N; 79^0^27’21” E; 1945 m. a.s.l.), Uttarakhand, India. Actively growing buds were used to initiate *in-vitro* cultures. The twigs were cleaned in 10% (v/v) Tween-20 (light detergent) solutions for 30 min before being rinsed multiple times with double distilled water. The twigs were then surface cleaned for 30 min in 0.1% mercuric chloride after being rinsed with sterile double distilled water. To initiate bud break, the branches were placed in flasks dipped in ½ MS basal medium without agar and housed in a growth chamber. Sprouted buds acquired in this manner were employed for *in-vitro* culture investigations.

### Culture conditions

All *in-vitro* studies used MS [49] medium supplemented with 3.0% (w/v) sucrose and 0.8% (w/v) agar (Hi-Media, Mumbai, India). The medium pH was adjusted to 5.8 ± 0.1 before it was dispensed in culture vessels and autoclaved at 121^0^ C at 15 lbs for 22 min. Unless otherwise noted, all cultures were incubated at 25°1 C for 16 h under cool-white fluorescent lights (PAR = 40Em-2 s-1).

### Explant sterilization and culture establishment

After 10–15 days of dipping, the axillary buds were collected from the twigs. These were then cleaned and surface sterilized in 70% ethanol for 30 s, 0.1% mercuric acid solution for 3 min, and rinsed several times in sterile distilled water before being grown in MS medium enriched with BA in combination with NAA. Explants were grown individually in initiation media for 30 days throughout the early phases (Fig. 2A). Following that, clumps of shoots were separated into single shoots and grown in multiplication media (Fig. 2B). To achieve a high multiplication rate, several concentrations of BA (1.0, 2.5, 5.0, and 10.0 µM) alone or in conjunction with NAA (0.01, 0.1, and 1.0 µM) were investigated (Table 1). The rate of multiplication, average shoots length, and numbers of leaves per shoot were calculated. Different combinations of BAP and NAA were tried. The best combination was identified and further tested for multiplication rate for 3 subcultures. Sub-culturing into the fresh medium was performed after 30 days.

### Rooting under in-vitro condition

In the first experiment, *in-vitro-*grown shoots were inoculated in half-strength MS basal medium supplemented with 1.5% (w/v) sucrose and 0.8% (w/v) agar with varying amounts of IBA (0.1, 1.0 and 10.0 µM) and NAA (0.1, 1.0, and 10.0 µM). Further, effect of the 7-day dark period (first 7 days of culture duration) on rooting performance was also tested (Table 6). The first experiment concluded that IBA with 7 days of dark treatment was best in terms of root induction and root length (Fig. 2C; D). However, to further optimize the concentration of IBA and dark period for optimal root induction in MM 104 rootstock, the effect of different dark duration (1, 5, 10 and 15 days) and different IBA (0.01, 0.1, 1.0, 5.0, 10.0 µM) concentration was tested (Table 2). Shoots were incubated at room temperature for 16 h under cool fluorescent lights (CFL = 40 mol m^-2^ s^-2^) at 25^0^ ± 2^0^ C inside the growth chamber for a total culture duration of 4 weeks. Dark treatment was not given to one set of cultures as they were placed directly in light for 4 weeks and used as control.

**Table 6:** Effect of two auxins (IBA and NAA) with different concentration and light/dark (7 days dark) treatment on rooting on MM 104 shoots

Treatments	Concentration (µM)	Root No.	Root length (cm)		Rooting %	
Light		Dark	Light	Dark	Light	Dark
T1	Control	0.00 (± 0.00)^e^	0.00(± 0.00)^e^	0.00(± 0.00)^d^	0.00(± 0.00)^f^	0.00(± 0.00)^d^
T2	0.1 IBA	4.33(± 0.11)^bc^	3.81(± 0.43)^a^	1.72(± 0.06)^a^	91.67(± 6.70)^a^	41.67(± 3.66)^b^
T3	1.0 IBA	5.31(± 0.15)^b^	2.01(± 0.04)^b^	1.27(± 0.04)^b^	75.00(± 4.32)^b^	45.83(± 4.12)^b^
T4	10.0 IBA	1.5 (± 0.02)^d^	0.75(± 0.01)^d^	0.68(± 0.00)^c^	25.00(± 2.54)^d^	45.83(± 4.00)^b^
T5	0.1 NAA	2.0 (± 0.18)^d^	1.50(± 0.01)c	1.77(± 0.06)^a^	12.50(± 1.60)^e^	50.00(± 3.88)^a^
T6	1.0 NAA	5.26(± 0.21)^b^	1.64(± 0.02)^c^	0.88(± 0.02)^c^	54.17(± 5.22)^c^	54.17(± 4.33)^a^
T7	10.0 NAA	3.98(± 0.09)^c^	1.10(± 0.02)^c^	0.61(± 0.01)^c^	58.33(± 6.44)^c^	29.17(± 2.11)^c^
T8	1.0 IBA + 1.0 NAA	7.48(± 0.60)^a^	1.08(± 0.02)^c^	0.76(± 0.01)^c^	58.33(± 6.230)^c^	50.00(± 4.64)^a^

Data are mean of three replicates each with eight explant, values in parenthesis are ± SE. For each parameter, values with different letters in superscript are significantly different (*p* < 0.05)

### Acclimatization

Different substrate types and combinations [S1 = vermiculite: perlite (1:1); S2 = vermiculite: perlite: sand (1:1:1); S3 = vermiculite: perlite: sand: soil (1:1:1:1); S4 = vermiculite: perlite: sand (1:1:1); S5 = vermiculite: perlite: sand: soil (2:2:1:1)] were utilized to determine the best conditions for acclimatizing *in-vitro*-grown plantlets. Before being treated with a systemic fungicide, rooted shoots were gently rinsed in running tap water to eliminate excess agar and sucrose residues, [Bavistin 0.1% (w/v) for 1 min], followed by distilled water rinse. Plantlets were then transferred into plastic pots (5 × 7.5 × 6.5 cm) filled with a soil, vermiculite, and perlite combination (1:2:2), ½ MS medium (without vitamins). Transparent polythene bags were used to seal the plantlets.

The polythene bags were progressively perforated and then eliminated after four weeks. In growth chamber settings, *in-vitro-*enhanced shoots’ growth performance was noticed (16 h light photoperiod at 25±1 °C) for 7 weeks and thereafter in a shade house for another 7 weeks (Fig. 2E). Thus, observations were made for a total of 14 weeks under *ex-vitro* conditions. Data on plantlet survival percentage and growth was recorded.

### Experimental design and statistical analysis

A randomized design was adopted in the present study. There were three copies of each therapy, each containing eight explants. Before analysis, the experiment’s percentage data underwent an arcsine transformation, and they were subsequently converted back to percentages for the tables’ interpretation [50]. The effect of different treatments, durations, and their interactions were enumerated by analysis of variance (ANOVA) and Fisher’s Least Significance Difference (LSD).

### Genetic stability analysis

An analysis of the genetic stability of *in-vitro* regenerated plants and the mother plant of MM104 was done using SCoT markers. Ten *in-vitro* regenerated plants from three different groups (15 plants in each group; T1, T2, and T3) were used to isolate DNA. DNA was obtained from 10 plants (ten from each group) and then samples were collected to determine genetic similarities. The technique described by [12] was employed to harvest DNA from juvenile leaves (500 mg).

According to Collard and Mackill, out of 36 SCoT primers [51], 12 primers (Table 6) were selected for the analysis [42], after preliminary screening. Ten ISSR primers (16–17 mer; University of British Columbia, Biotechnology Laboratory, Vancouver, Canada) after screening, were chosen (primer set 9) for PCR amplification [52–54]. A total volume of 25 µl was used for the PCR amplification, which included 2.5 µl of 10X PCR buffer containing 15 mM MgCl2, 0.2 mM dNTPs, 1unit Taq Polymerase (Sigma, USA), 20ng of genomic DNA, and 20ng of Primer (Integrated DNA Technologies Inc., India). The following reaction conditions were used for performing PCR: one cycle of DNA denaturation at 94 °C for 4 min, 38 cycles of 30 s denaturation at 94 °C, annealing at Tm°C (estimated for every primer), and 1 min of extension at 72 °C, with an 8-minute final extension at 72 °C.

Using the UV light-gel documentation gystem, agarose gel electrophoresis was used to separate the amplified results of the ISSR and SCoT analyses (UVP Ltd, Cambridge, UK). For analysis, only bands that were visible, firmly stained, and reproducible were chosen. The binary character was used to assess the presence or absence of bands, and the similarity coefficient was calculated.

## Data Availability

The data presented in this study are available at a reasonable request from the corresponding author.
